# Korean Red Ginseng exerts anti-inflammatory and autophagy-promoting activities in aged mice

**DOI:** 10.1016/j.jgr.2021.03.009

**Published:** 2021-04-06

**Authors:** Jin Kyeong Kim, Kon Kuk Shin, Haeyeop Kim, Yo Han Hong, Wooram Choi, Yi-Seong Kwak, Chang-Kyun Han, Sun Hee Hyun, Jae Youl Cho

**Affiliations:** a Department of Integrative Biotechnology, Sungkyunkwan University, Suwon, Republic of Korea; b R&D Headquarters, Korea Ginseng Corporation, Daejeon, Republic of Korea

**Keywords:** Korean Red Ginseng (KRG), anti-inflammatory effect, autophagy, aging, KRG, Korean Red Ginseng, RT-PCR, reverse transcription polymerase chain reaction, TNF-α, tumor necrosis factor-α, IL, interleukin, NF-κB, nuclear factor-kappa B, AP-1, activator protein-1, ATG, autophagy-related gene, MCP-1, monocyte chemoattractant protein-1

## Abstract

**Background:**

Korean Red Ginseng (KRG) is a traditional herb that has several beneficial properties including anti-aging, anti-inflammatory, and autophagy regulatory effects. However, the mechanisms of these effects are not well understood. In this report, the underlying mechanisms of anti-inflammatory and autophagy-promoting effects were investigated in aged mice treated with KRG-water extract (WE) over a long period.

**Methods:**

The mechanisms of anti-inflammatory and autophagy-promoting activities of KRG-WE were evaluated in kidney, lung, liver, stomach, and colon of aged mice using semi-quantitative reverse transcription polymerase chain reaction (RT-PCR), quantitative RT-PCR (qRT-PCR), and western blot analysis.

**Results:**

KRG-WE significantly suppressed the mRNA expression levels of inflammation-related genes such as interleukin (IL)-1β, IL-8, tumor necrosis factor (TNF)-α, monocyte chemoattractant protein-1 **(**MCP-1), and IL-6 in kidney, lung, liver, stomach, and colon of the aged mice. Furthermore, KRG-WE downregulated the expression of transcription factors and their protein levels associated with inflammation in lung and kidney of aged mice. KRG-WE also increased the expression of autophagy-related genes and their protein levels in colon, liver, and stomach.

**Conclusion:**

The results suggest that KRG can suppress inflammatory responses and recover autophagy activity in aged mice.

## Graphical abstract

**Figure:**
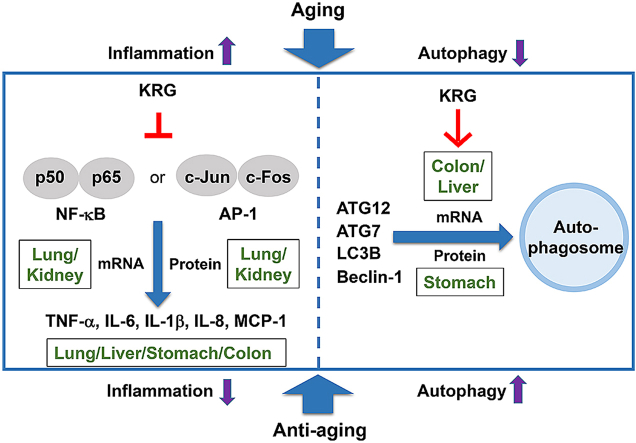

## 1 Introduction

Inflammation in aging is associated with an increased rate of various degenerative diseases including Parkinson’s disease, osteoarthritis, Huntington’s disease, and Alzheimer’s disease [[1], [2], [3]]. These disorders are associated with chronic inflammation. Patients with age-dependent diseases show a chronic inflammatory state such as increase of inflammatory cells and higher pro-inflammatory cytokine levels [4,5]. Age-related disorders in tissues are harmful for important organs such as kidney, lung, and liver. The transcriptional ability becomes dysfunctional during aging. In particular, aging regulates epigenetic modifications, causing changes in gene expression [6,7].

Autophagy is a cytoprotective mechanism that induces degradation and recycling of cytoplasmic organelles to provide new nutrients and energy [8,9]. A dysfunction in autophagic activity due to age contributes to accumulation of damaged intracellular organelles that result in imbalance of cellular homeostasis and loss of function in aging [10,11], which can mediate organ damage affecting the liver, lung, kidney, and nervous system [12,13]. Furthermore, defective autophagy is associated with common age-related diseases [14,15].

Panax ginseng is a traditional herb used as medicine in Korea and China for thousands of years [16,17]. In previous studies, Panax ginseng was reported to exert numerous beneficial effects such as anti-diabetic, anti-aging, anti-inflammatory, anti-tumor, and autophagy regulation [[18], [19], [20], [21], [22]]. Because chronic inflammation and decrease of autophagic activity are associated with aging, the effects of Panax ginseng on aging were examined in the present study [23,24]. Therefore, the molecular mechanisms of anti-inflammatory and autophagy regulating effects caused by Korean Red Ginseng (KRG) were investigated in aged mice.

## 2 Materials and methods

### 2.1 Materials

KRG-water extract (KRG-WE: Hongsamjeong) was obtained from Korea Ginseng Corp. (Daejeon, Korea), and the major components of KRG-WE are shown in Supplementary Table 1 as reported previously [25,26]. Two-month-old C57BL/6J male mice (young mice) and 17-month-old C57BL/6J male mice (aged mice) were obtained from Dae Han Bio Link Co., Ltd. (Osong, Korea). Metformin and sodium dodecyl sulfate (SDS) were acquired from Sigma-Aldrich (St. Louis, MO, USA). The antibodies against p50, p65, c-Jun, c-Fos, autophagy related 7 (ATG7), ATG12, light chain 3B (LC3B), beclin-1, and β-actin used for immunoblotting analysis were obtained from Cell Signaling Technology (Beverly, MA, USA).

### 2.2 Animals and treatment dose

C57BL/6J mice were housed in a standard plastic cage under 12-h light/12-h dark cycles. Mice were randomly divided into the following four groups: (I) young mice (2 month-old), (II) aged mice (17 month-old) (III) aged mice treated with KRG-WE 200 mg/kg/day, and (IV) aged mice treated with metformin 200 mg/kg/day. Mice (7 mice/group) were orally treated with KRG-WE (200 mg/kg), or metformin (200 mg/kg) once a day for 30 days. Dose of KRG-WE was decided by previous animal experiments carried out with crude extracts [27,28]. Metformin as anti-aging drug was used according to previous report [29]. Animal care followed the guidelines of the Institutional Animal Care and Use Committee at Sungkyunkawn University (Approval number: 2018-10-16-1).

### 2.3 Semi-quantitative RT-PCR and qRT-PCR

Total RNA was isolated from animal tissues using TRIzol Reagent following the manufacturer’s instructions. After measuring the total amount of RNA, cDNAs were synthesized from total RNA (1 μg) using MMLV RTase (SuperBio, Daejeon, Korea). Quantification of mRNA expression was performed using semi-quantitative reverse transcription polymerase chain reaction (RT-PCR) or quantitative RT-PCR (qRT-PCR) as previously described [30]. All primer sequences are listed in Supplementary Table 2.

### 2.4 Immunoblotting analysis

Whole lysates were extracted from animal tissues. Lysates were prepared using lysis buffer. Protein targets were detected using the specific antibodies. Immunoblotting analysis was conducted as previously reported [31].

### 2.5 Statistical analyses

All data in the present study are presented as mean ± standard deviation (SD). To compare the data, Mann-Whitney tests was utilized. All statistical tests were performed using the computer program SPSS (version 26, SPSS Inc., Chicago, IL, USA), and a p-value < 0.05 was considered statistically significant.

## 3 Results and discussion

### 3.1 KRG-WE decreased the mRNA expression levels of inflammatory cytokines in lung, kidney, liver, stomach, and colon

**Fig. 1:**
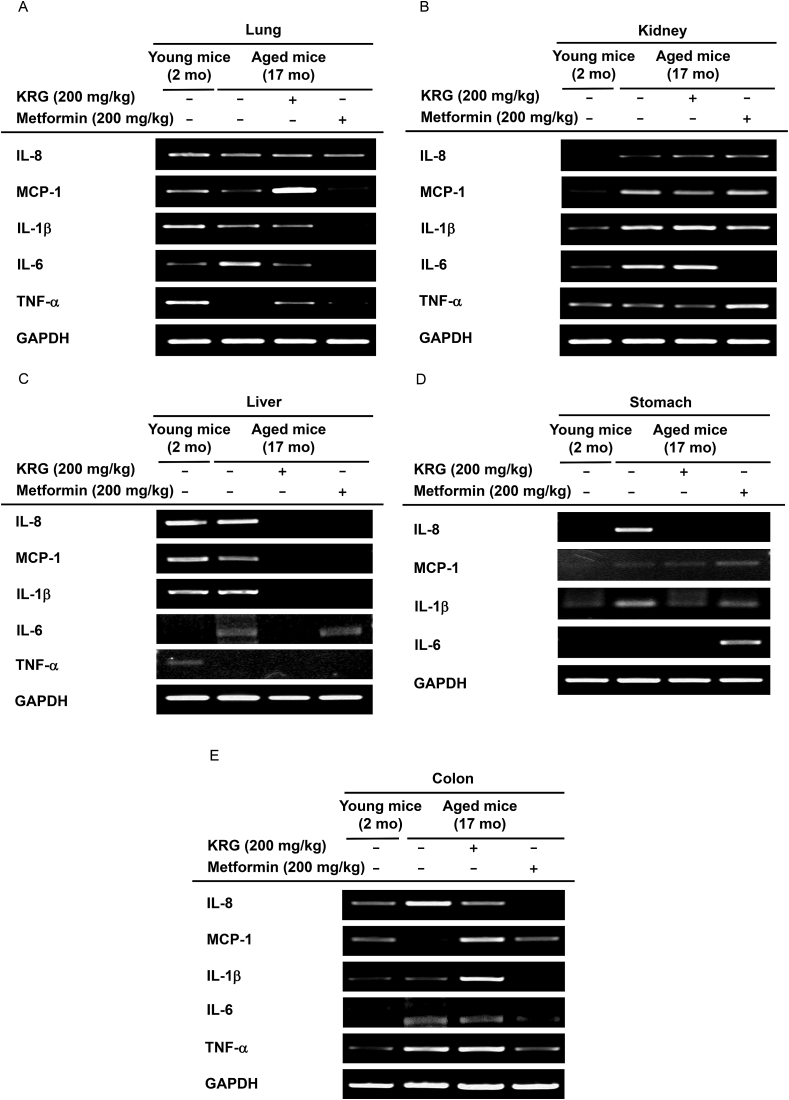
Suppressive effects of KRG-WE on mRNA expression levels of inflammatory cytokines in lung, kidney, liver, stomach, and colon. (A–E) KRG-WE (200 mg/kg) was orally administered to aged mice. The IL-1β, TNF-α, IL-8, MCP-1, and IL-6 mRNA expression levels in lung, kidney, liver, stomach, and colon were measured using semi-quantitative RT-PCR.

Aging induces chronic inflammatory activity evidenced by increased expression of inflammation-related genes including tumor necrosis factor (TNF)-α, interleukin (IL)-6, monocyte chemoattractant protein-1 **(**MCP-1), IL-1β, and IL-8 [32,33]. To examine whether KRG-WE can inhibit the inflammatory activities in lung, kidney, liver, stomach, and colon of aged mice, the mRNA expression levels of inflammation related genes were evaluated. In lung, chronic inflammation upregulates the platelet-activating factor receptors on the surface of epithelial cells and can induce bacterial adhesion and accumulation in the aged lung [34]. Although the IL-6 expression level in lung was increased in the aged mice, KRG-WE (200 mg/kg) suppressed IL-6 expression in aged mice (Fig. 1A). Metformin is a control drug that has been used to treat diabetes and is associated with aging-related activities [35,36]. Metformin (200 mg/kg) decreased IL-6 expression similar to KRG-WE (200 mg/kg). Repeat tissue inflammation accelerates the aging process in the kidney [37]. IL-8, MCP-1, IL-1β, and IL-6 expression levels were increased in the kidney of aged mice, while KRG-WE (200 mg/kg) downregulated MCP-1 expression (Fig. 1B). The incidence of liver diseases accompanied by inflammation resulting from damaged hepatic cells increases with age [38]. Although IL-1β and IL-6 expression levels were increased in the liver of aged mice, KRG-WE (200 mg/kg) and control drug metformin (200 mg/kg) downregulated IL-1β and IL-6 expression in aged mice (Fig. 1C). Gastritis is associated with aging of the stomach and is characterized by chronic inflammation [39]. Although the IL-1β and IL-8 expression levels in stomach were increased in the aged mice, KRG-WE (200 mg/kg) and control drug metformin (200 mg/kg) downregulated the IL-1β and IL-8 expression in aged mice (Fig. 1D). Chronic inﬂammation including intestinal bowel disease, which is common in older people, can provoke tumorigenic responses [40]. In the colon, IL-8, IL-6, and TNF-α expression levels were increased in the aged mice, while KRG-WE (200 mg/kg) and control drug metformin (200 mg/kg) downregulated IL-6 and IL-8 expression in aged mice (Fig. 1E). These data suggest that KRG-WE has anti-inflammatory effect by suppressing mRNA expression of inflammatory cytokines including IL-8, MCP-1, IL-1β, IL-6, and TNF-α in lung, kidney, liver, stomach, and colon of aged mice.

### 3.2 KRG-WE downregulated the mRNA expression levels of transcriptional factor subunits associated with inflammation in lung, kidney, and colon

**Fig. 2:**
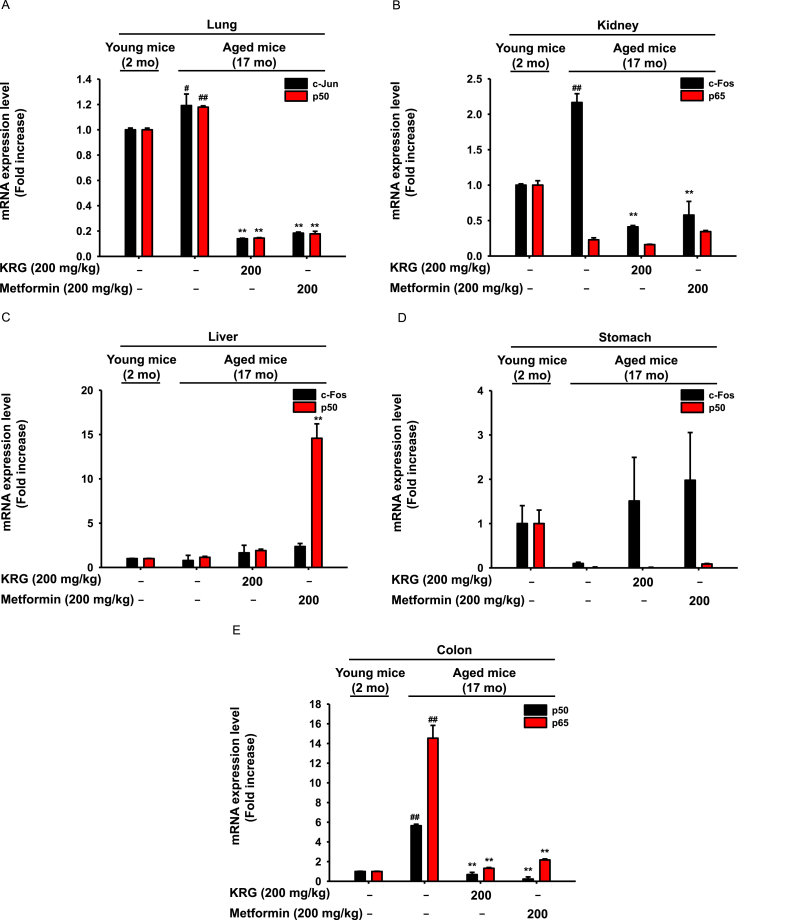
Inhibitory effects of KRG-WE on mRNA expression level of transcription factor NF-κB or AP-1. (A–E) The mRNA expression levels of p50, p65, c-Fos, and c-Jun that are subunits of NF-κB or AP-1 were measured in lung, kidney, liver, stomach, and colon using qRT-PCR. #P < 0.05 and ##P < 0.01 compared with the normal group; ∗ P < 0.05 and ∗∗ P < 0.01 compared with the control group.

The expression of inflammatory cytokines is regulated by transcriptional factors such as nuclear factor (NF)-κB and activator protein (AP)-1 [41,42]. The NF-κB family is composed of five structurally similar subunits, p50, p52, p65, Rel B, and c-Rel, which regulate the expression of target genes associated with inflammation by binding heterodimers or homodimers [43,44]. The AP-1 is a dimeric transcription factor comprised of c-Fos and c-Jun and mediates inflammation-related genes [45,46]. To evaluate the effects of KRG on transcriptional factors associated with inflammation, the mRNA levels of transcriptional factor subunits were analyzed. RT-PCR results showed that expression levels of c-Jun and p50 were increased in aged mice, and KRG-WE (200 mg/kg) inhibited the mRNA expression of c-JUN and p50 (Fig. 2A). In kidney, mRNA expression level of the AP-1 subunit c-Fos was upregulated in aged mice, while KRG-WE (200 mg/kg) inhibited the mRNA expression of c-Fos (Fig. 2B). In liver, expression levels of transcription factor subunits p50 and c-Fos did not differ between young and aged mice (Fig. 2C). In stomach, KRG-WE did not decrease the expression levels of p50 and p65 in aged mice (Fig. 2D). In colon, expression levels of p50 and p65 were increased in aged mice, while KRG-WE (200 mg/kg) suppressed the protein expression of p50 and p65 (Fig. 2E). Taken together, these results indicate that KRG-WE suppresses mRNA expression of transcription factors associated with inflammation, such as c-Jun, c-Fos, p50, and p65, in lung and kidney of aged mice.

### 3.3 KRG-WE suppressed the protein levels of transcriptional factor subunits associated with inflammation in lung, kidney, stomach, and liver

**Fig. 3:**
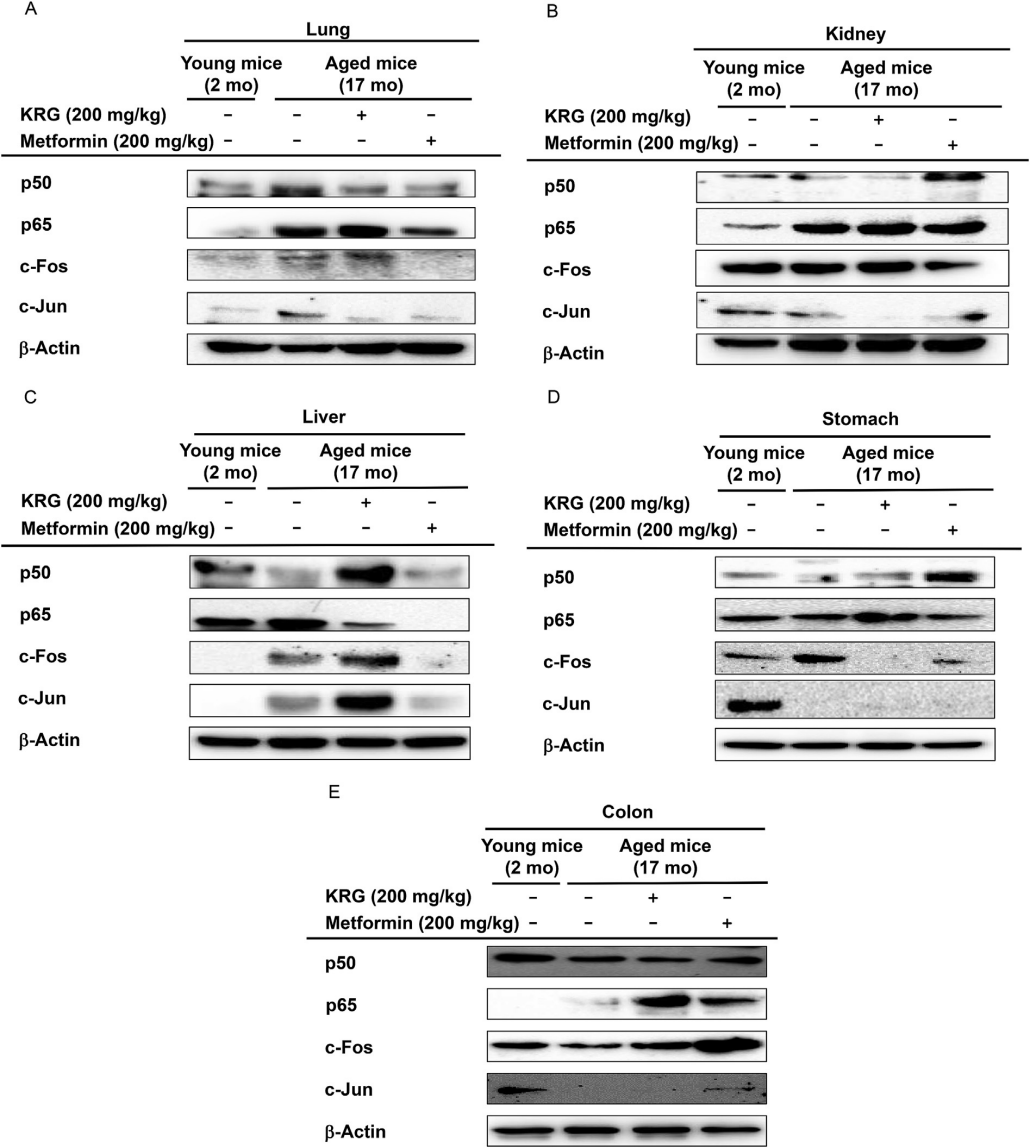
KRG-WE exerted anti-inflammatory effects by regulating the protein expression levels of transcription factors NF-κB and AP-1. (A–E) The protein expression levels of p50, p65, c-Fos, and c-Jun, subunits of NF-κB or AP-1, were evaluated in lung, kidney, liver, stomach, and colon of mice using western blot analysis.

Because KRG-WE affected the expression of transcription factors at the mRNA level, the protein expression level of transcription factors associated with inflammation was evaluated using western blot analysis. In lung of aged mice, KRG-WE (200 mg/kg) decreased the p50 and c-Jun protein expression levels (Fig. 3A). The p50 protein expression level was increased in kidney of aged mice, but KRG-WE (200 mg/kg) inhibited the p50 expression level (Fig. 3B). KRG-WE (200 mg/kg) decreased p65 protein expression level in liver of aged mice (Fig. 3C). KRG-WE (200 mg/kg) decreased c-Fos protein expression level in the stomach of aged mice (Fig. 3D). The protein expression levels of NF-κB and AP-1 subunits in the colon did not differ between young and aged mice (Fig. 3E). Similar to the results of mRNA expression levels, these data indicate that KRG-WE downregulated the protein expression levels of transcription factors associated with inflammation in lung and kidney of aged mice.

### 3.4 KRG-WE increased the mRNA levels of autophagy-related genes in stomach, kidney, lung, liver, and colon

**Fig. 4:**
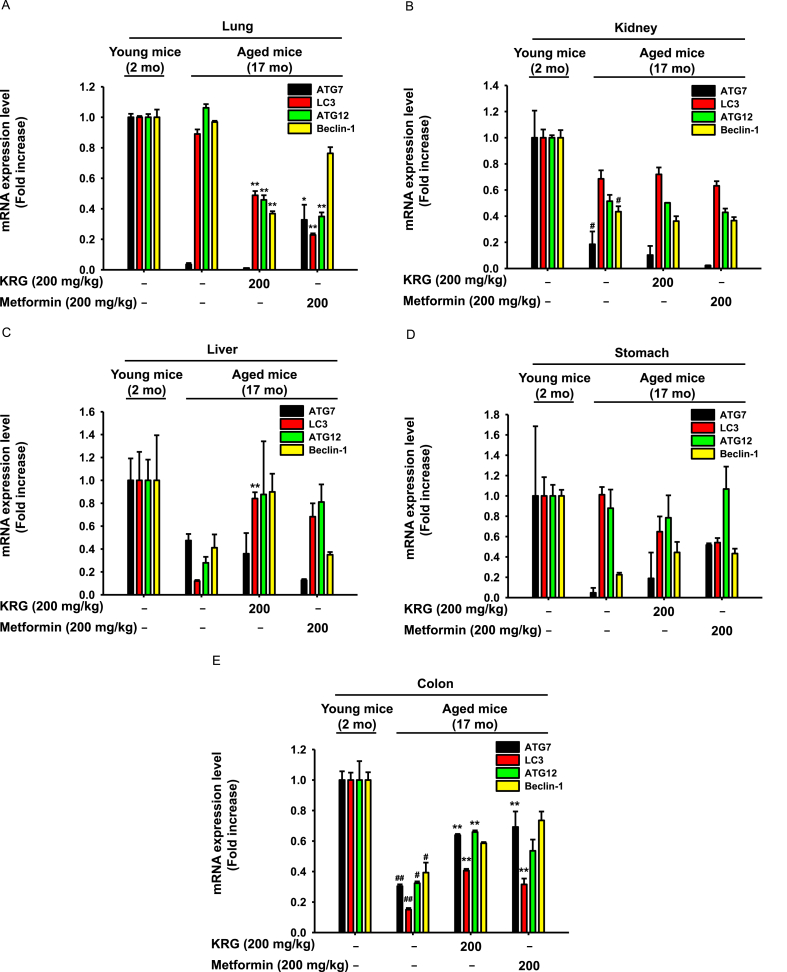
Autophagy-promoting effects of KRG-WE by increasing the mRNA expression levels of autophagy-related genes. (A–E) The mRNA expression levels of ATG7, ATG12, LC3B, and beclin-1 were measured using qRT-PCR. #P < 0.05 and ##P < 0.01 compared with the normal group; ∗ P < 0.05 and ∗∗ P < 0.01 compared with the control group.

In several studies, autophagy in age-related diseases was associated with genetic alterations of autophagy-related proteins [47,48]. Therefore, the autophagy-promoting activity of KRG-WE was assessed in the present study by determining the mRNA levels of autophagy-related genes. ATG7, ATG12, LC3B, and beclin-1 are autophagy-related genes that are essential for formation of the autophagosome [[49], [50], [51]]. In lung cells, autophagy represents a protective response to injury resulting from exposure to stress stimuli such as hypoxia, oxidants, inflammation, and aging [52]. The mRNA expression levels of ATG7 and LC3B were decreased in the lung of aged mice but were unchanged by KRG-WE (200 mg/kg) treatment (Fig. 4A). Autophagic activity in kidney diseases regulates immune responses that decrease with age. The mRNA expression levels of ATG12, ATG7, LC3B, and beclin-1 were decreased in kidney of aged mice, but KRG-WE (200 mg/kg) did not increase the mRNA expression level (Fig. 4B). In the liver, autophagy is a major process that exerts cytoprotective effects against prolonged ischemia and reperfusion injury [53]. Although the mRNA expression levels of ATG12, ATG7, LC3B, and LC3B were decreased in the liver of aged mice, KRG-WE (200 mg/kg) recovered the expression of ATG12, LC3B, and beclin-1 (Fig. 4C). Autophagy recovers injury of gastric epithelial cells induced by oxidative stress or aging [54]. The mRNA expression levels of ATG7 and beclin-1 were decreased in the stomach of aged mice, but KRG-WE (200 mg/kg) recovered these levels (Fig. 4D). Essential energy sources in the colon have been identified as autophagy mediators and might exert diverse functions of energy metabolism during aging [55]. Although the mRNA expression level of ATG12, ATG7, LC3B, and beclin-1 were decreased in aged mice, KRG-WE (200 mg/kg) recovered the expression levels in colon (Fig. 4E). Overall, KRG-WE upregulated the mRNA expression levels of autophagy-related genes such as LC3B, ATG7, ATG12, and beclin-1 in liver and colon of aged mice.

### 3.5 KRG-WE increased the protein levels of autophagy-related genes in stomach and lung

**Fig. 5:**
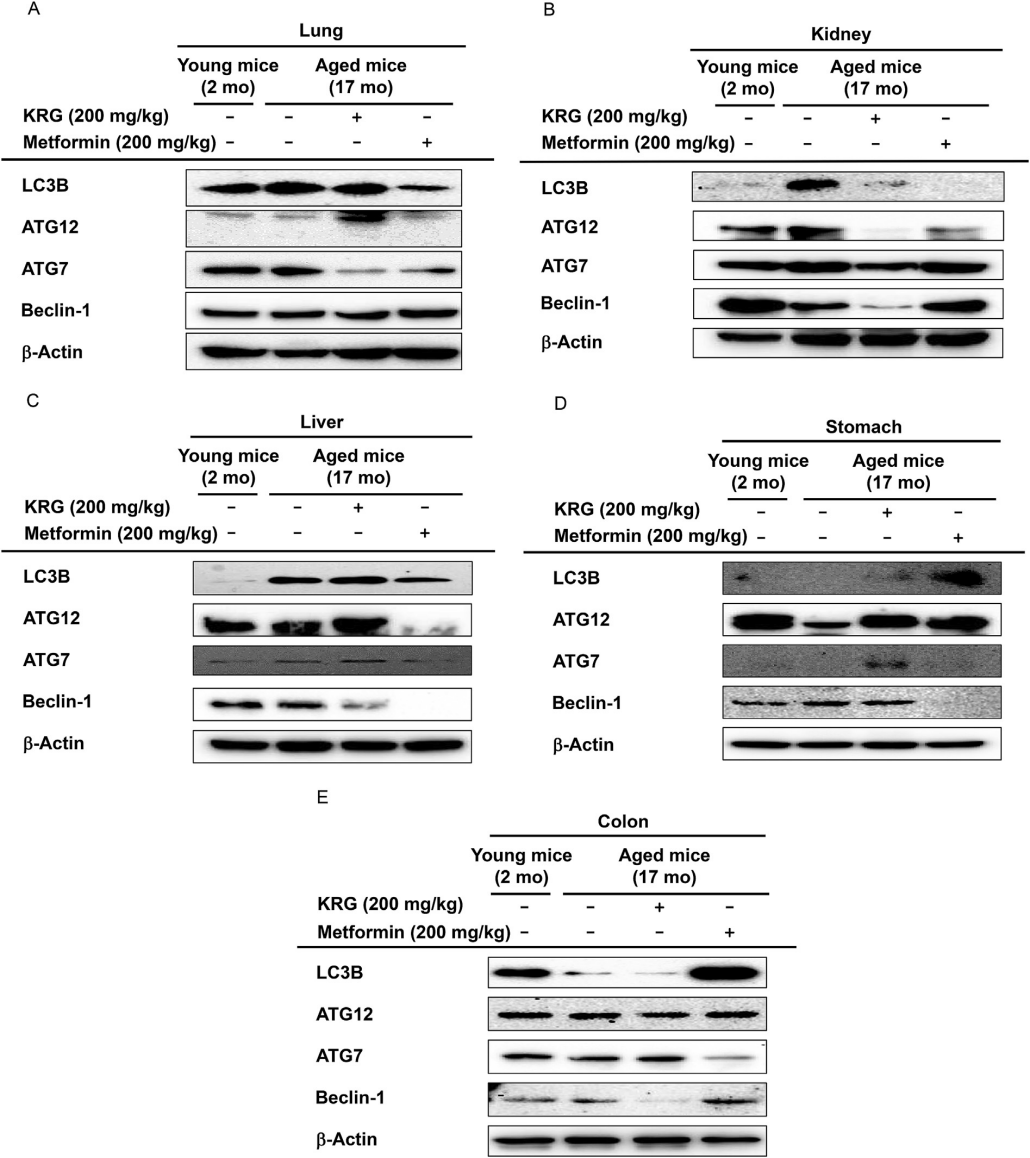
Autophagy-promoting effects of KRG-WE by increasing the protein expression levels of autophagy-related genes. (A–E) The protein expression levels of ATG7, ATG12, LC3B, and beclin-1 were measured using western blot analysis.

To explore whether KRG regulates the protein synthesis of autophagy-related genes, the effects of KRG-WE were evaluated at the protein level. In lung, the protein level of ATG12 was downregulated in aged mice compared with young mice, but KRG-WE (200 mg/kg) increased ATG12 expression level in aged mice (Fig. 5A). In kidney, the expression level of Beclin-1 was decreased in aged mice compared with young mice, and KRG-WE (200 mg/kg) did not recover the expression (Fig. 5B). In liver, KRG-WE (200 mg/kg) recovered ATG12 and ATG7 protein levels that were decreased in aged mice (Fig. 5C). In stomach, protein levels of LC3B, ATG12, and ATG7 were downregulated in aged mice, but KRG-WE (200 mg/kg) recovered the expression levels (Fig. 5D). In colon, the expression level of LC3B was downregulated in aged mice, and KRG-WE (200 mg/kg) did not recover the expression (Fig. 5E). Taken together, these results suggest that KRG exerts autophagy-promoting activity in the stomach by decreasing the expression of autophagy-related genes such as ATG7, ATG12, and LC3B.

## 4 Conclusion

**Fig. 6:**
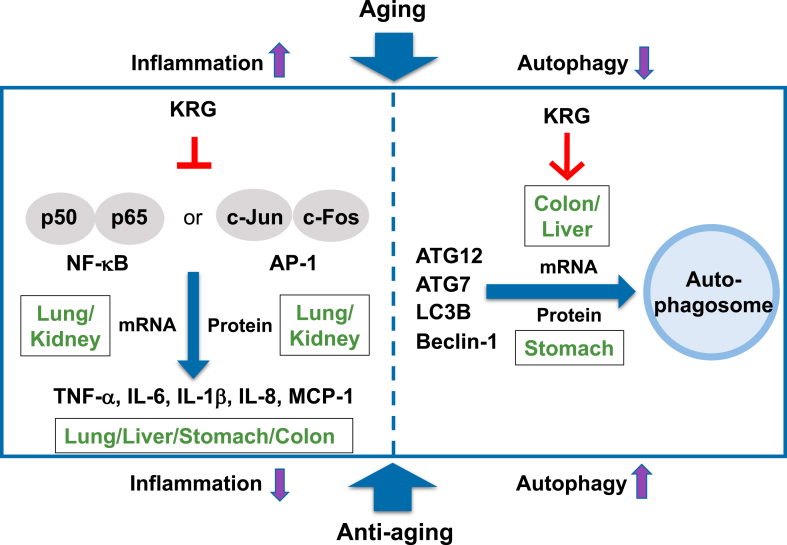
Summary of anti-inflammatory and autophagy-promoting mechanisms of KRG-WE in lung, kidney, liver, stomach, and colon of aged mice.

In summary, the mechanisms of anti-inflammatory and autophagy-promoting effects of KRG in aged mice were investigated. Administration of KRG-WE for 8 weeks significantly suppressed the mRNA expression levels of inflammation-related genes such as IL-1β, TNF-α, IL-8, MCP-1, and IL-6 in lung, kidney, liver, stomach, and colon of aged mice. Furthermore, KRG-WE suppressed the expression of transcription factors including NF-κB and AP-1 that stimulate the expression of inflammatory cytokines in lung and kidney. KRG-WE (200 mg/kg) also inhibited NF-κB and AP-1 protein levels, specifically in lung and kidney. These results indicate that the inflammatory response can be inhibited by KRG in lung, kidney, liver, and stomach of aged mice. KRG-WE promoted autophagy activity by increasing the expression level of autophagy-related genes such as ATG7, ATG12, LC3B, and beclin-1 in liver and colon of aged mice. In particular, KRG-WE (200 mg/kg) recovered ATG7, ATG12, and LC3B protein expression levels in the stomach of aged mice. These data indicate that KRG exerts anti-inflammatory and autophagy-promoting activities especially in lung, liver, and stomach of aged mice as summarized in Fig. 6. Collectively, these results suggest that KRG can be used as an herbal medicine with anti-inflammatory and autophagy-promoting effects in the elderly.

## Author contributions

JKK, S.H.H, and JYC conceived and designed the experiments; JKK, KKS, HK, YHH, and WC performed the experiments; JKK, KKS, HK, YHH, WC, Y.-S.K., C.-K.H., S.H.H, and JYC analyzed the data; JKK, S.H.H, and JYC wrote the manuscript.

## Declaration of competing interest

The authors declare no conflicts of interest. All authors listed have read and approved the submitted manuscript. This manuscript has not been submitted to or published in any journal and is not being considered for publication elsewhere.

## References

[1] McGeer P.L., McGeer E.G. (2004). Inflammation and the degenerative diseases of aging. Annals of the New York Academy of Sciences.

[2] Greene M.A., Loeser R.F. (2015). Aging-related inflammation in osteoarthritis. Osteoarthritis and Cartilage.

[3] Holmes C., Cunningham C., Zotova E., Woolford J., Dean C., Su Kerr, Culliford D., Perry V. (2009). Systemic inflammation and disease progression in Alzheimer disease. Neurology.

[4] Sarkar D., Fisher P.B. (2006). Molecular mechanisms of aging-associated inflammation. Cancer Letters.

[5] Michaud M., Balardy L., Moulis G., Gaudin C., Peyrot C., Vellas B., Cesari M., Nourhashemi F. (2013). Proinflammatory cytokines, aging, and age-related diseases. Journal of the American Medical Directors Association.

[6] Brunet A., Berger S.L. (2014). Epigenetics of aging and aging-related disease. Journals of Gerontology Series A: Biomedical Sciences and Medical Sciences.

[7] Donmez G., Guarente L. (2010). Aging and disease: connections to sirtuins. Aging Cell.

[8] Rubinsztein D.C., Mariño G., Kroemer G. (2011). Autophagy and aging. Cell.

[9] Zhang C., Cuervo A.M. (2008). Restoration of chaperone-mediated autophagy in aging liver improves cellular maintenance and hepatic function. Nature Medicine.

[10] Cheon S.Y., Kim H., Rubinsztein D.C., Lee J.E. (2019). Autophagy, cellular aging and Age-related human diseases. Experimental Neurobiology.

[11] Martinez-Lopez N., Athonvarangkul D., Singh R. (2015). Autophagy and aging. Longevity Genes.

[12] Jiang M., Liu K., Luo J., Dong Z. (2010). Autophagy is a renoprotective mechanism during in vitro hypoxia and in vivo ischemia-reperfusion injury. The American Journal of Pathology.

[13] Lenoir O., Tharaux P.-L., Huber T.B. (2016). Autophagy in kidney disease and aging: lessons from rodent models. Kidney International.

[14] Massey A.C., Zhang C., Cuervo A.M. (2006). Chaperone-mediated autophagy in aging and disease. Current Topics in Developmental Biology.

[15] Cuervo A.M., Wong E. (2014). Chaperone-mediated autophagy: roles in disease and aging. Cell Research.

[16] Yun T.K. (2001). Brief introduction of Panax ginseng CA Meyer. Journal of Korean Medical Science.

[17] Lee S.M., Bae B.-S., Park H.-W., Ahn N.-G., Cho B.-G., Cho Y.-L., Kwak Y.-S. (2015). Characterization of Korean red ginseng (Panax ginseng Meyer): history, preparation method, and chemical composition. Journal of Ginseng Research.

[18] Lee D.C., Lau A.S. (2011). Effects of Panax ginseng on tumor necrosis factor-α-mediated inflammation: a mini-review. Molecules.

[19] Yoo H.-S., Kim J.M., Jo E., Cho C.-K., Lee S.-Y., Kang H.S., Lee M.-G., Yang P.-Y., Jang I.-S. (2017). Modified Panax ginseng extract regulates autophagy by AMPK signaling in A549 human lung cancer cells. Oncology Reports.

[20] Qomaladewi N.P., Kim M.-Y., Cho J.Y. (2019). Autophagy and its regulation by ginseng components. Journal of Ginseng Research.

[21] Kim J.H., Yi Y.-S., Kim M.-Y., Cho J.Y. (2017). Role of ginsenosides, the main active components of Panax ginseng, in inflammatory responses and diseases. Journal of Ginseng Research.

[22] Lee J.-I., Park K.S., Cho I.-H. (2019). Panax ginseng: a candidate herbal medicine for autoimmune disease. Journal of Ginseng Research.

[23] Chung H.Y., Kim D.H., Lee E.K., Chung K.W., Chung S., Lee B., Seo A.Y., Chung J.H., Jung Y.S., Im E. (2019). Redefining chronic inflammation in aging and age-related diseases: proposal of the senoinflammation concept. Aging and Disease.

[24] Ren J., Zhang Y. (2018). Targeting autophagy in aging and aging-related cardiovascular diseases. Trends in Pharmacological Sciences.

[25] Lee J., Park J., Lee Y.Y., Lee Y. (2020). Comparative transcriptome analysis of the protective effects of Korean Red Ginseng against the influence of bisphenol A in the liver and uterus of ovariectomized mice. J Ginseng Res.

[26] Park J.G., Son Y.J., Aravinthan A., Kim J.H., Cho J.Y. (2016). Korean Red Ginseng water extract arrests growth of xenografted lymphoma cells. J Ginseng Res.

[27] Kim J.H., Park J.G., Hong Y.H., Shin K.K., Kim J.K., Kim Y.D., Yoon K.D., Kim K.H., Yoo B.C., Sung G.H. (2021). Sauropus brevipes ethanol extract negatively regulates inflammatory responses in vivo and in vitro by targeting Src, Syk and IRAK1. Pharm Biol.

[28] Park S.H., Oh J., Jo M., Kim J.K., Kim D.S., Kim H.G., Yoon K., Yang Y., Geum J.H., Kim J.E. (2020). Water extract of Lotus leaf alleviates dexamethasone-induced muscle atrophy via regulating protein metabolism-related pathways in mice. Molecules.

[29] Zhu X., Shen J., Feng S., Huang C., Liu Z., Sun Y.E., Liu H. (2020). Metformin improves cognition of aged mice by promoting cerebral angiogenesis and neurogenesis. Aging (Albany NY).

[30] Choi E., Kim E., Kim J.H., Yoon K., Kim S., Lee J., Cho J.Y. (2019). AKT1-targeted proapoptotic activity of compound K in human breast cancer cells. Journal of Ginseng Research.

[31] Han S.Y., Yi Y.-S., Jeong S.-G., Hong Y.H., Choi K.J., Hossain M.A., Hwang H., Rho H.S., Lee J., Kim J.-H. (2018). Ethanol extract of lilium bulbs plays an anti-inflammatory role by targeting the IKK α/β-Mediated NF-κ B pathway in macrophages. The American Journal of Chinese Medicine.

[32] Morley J.E., Baumgartner R.N. (2004). Cytokine-related aging process. The Journals of Gerontology Series A: Biological Sciences and Medical Sciences.

[33] Bruunsgaard H., Pedersen M., Pedersen B.K. (2001). Aging and proinflammatory cytokines. Current Opinion in Hematology.

[34] Cho S.J., Stout-Delgado H.W. (2020). Aging and lung disease. Annual Review of Physiology.

[35] Hunto S.T., Kim H.G., Baek K.-S., Jeong D., Kim E., Kim J.H., Cho J.Y. (2020). Loratadine, an antihistamine drug, exhibits anti-inflammatory activity through suppression of the NF-kB pathway. Biochemical Pharmacology.

[36] Barzilai N., Crandall J.P., Kritchevsky S.B., Espeland M.A. (2016). Metformin as a tool to target aging. Cell Metabolism.

[37] Nitta K., Okada K., Yanai M., Takahashi S. (2013). Aging and chronic kidney disease. Kidney and Blood Pressure Research.

[38] Kim H., Kisseleva T., Brenner D.A. (2015). Aging and liver disease. Current Opinion in Gastroenterology.

[39] Sonnenberg A., Genta R.M. (2015). Changes in the gastric mucosa with aging. Clinical Gastroenterology and Hepatology.

[40] Motilva V., García-Mauriño S., Talero E., Illanes M. (2011). New paradigms in chronic intestinal inflammation and colon cancer: role of melatonin. Journal of Pineal Research.

[41] Cao S., Zhang X., Edwards J.P., Mosser D.M. (2006). NF-κB1 (p50) homodimers differentially regulate pro-and anti-inflammatory cytokines in macrophages. Journal of Biological Chemistry.

[42] Jedinak A., Dudhgaonkar S., Wu Q.-l., Simon J., Sliva D. (2011). Anti-inflammatory activity of edible oyster mushroom is mediated through the inhibition of NF-κB and AP-1 signaling. Nutrition Journal.

[43] Liu T., Zhang L., Joo D., Sun S.-C. (2017). NF-κB signaling in inflammation. Signal Transduction and Targeted Therapy.

[44] Baeuerle P.A., Baichwal V.R. (1997). NF-kB as a frequent target for immunosuppressive and anti-inflammatory molecules. Advances in Immunology.

[45] Schonthaler H.B., Guinea-Viniegra J., Wagner E.F. (2011). Targeting inflammation by modulating the Jun/AP-1 pathway. Annals of the Rheumatic Diseases.

[46] Matthews C.P., Colburn N.H., Young M.R. (2007). AP-1 a target for cancer prevention. Current Cancer Drug Targets.

[47] Kimura T., Isaka Y., Yoshimori T. (2017). Autophagy and kidney inflammation. Autophagy.

[48] Omata Y., Lim Y.-M., Akao Y., Tsuda L. (2014). Age-induced reduction of autophagy-related gene expression is associated with onset of Alzheimer’s disease. American Journal of Neurodegenerative Disease.

[49] Arakawa S., Honda S., Yamaguchi H., Shimizu S. (2017). Molecular mechanisms and physiological roles of Atg5/Atg7-independent alternative autophagy. Proceedings of the Japan Academy, Series B.

[50] Mai S., Muster B., Bereiter-Hahn J., Jendrach M. (2012). Autophagy proteins LC3B, ATG5 and ATG12 participate in quality control after mitochondrial damage and influence lifespan. Autophagy.

[51] Schaaf M.B., Keulers T.G., Vooijs M.A., Rouschop K.M. (2016). LC3/GABARAP family proteins: autophagy-(un) related functions. The FASEB Journal.

[52] Ryter S.W., Choi A.M. (2010). Autophagy in the lung. Proceedings of the American Thoracic Society.

[53] Wang J.H., Ahn I.S., Fischer T.D., Byeon J.I., Dunn W.A., Behrns K.E., Leeuwenburgh C., Kim J.S. (2011). Autophagy suppresses age-dependent ischemia and reperfusion injury in livers of mice. Gastroenterology.

[54] Chang W., Bai J., Tian S., Ma M., Li W., Yin Y., Deng R., Cui J., Li J., Wang G. (2017). Autophagy protects gastric mucosal epithelial cells from ethanol-induced oxidative damage via mTOR signaling pathway. Experimental Biology and Medicine.

[55] Schroeder S., Zimmermann A., Carmona-Gutierrez D., Eisenberg T., Ruckenstuhl C., Andryushkova A., Pendl T., Harger A., Madeo F. (2014). Metabolites in aging and autophagy. Microbial Cell.

