# Supplementary material for: Korean Red Ginseng exerts anti-inflammatory and autophagy-promoting activities in aged mice
Source: J Ginseng Res. 2021 Apr 6;45(6):717–25. doi: 10.1016/j.jgr.2021.03.009 (PMC8569327; doi:10.1016/j.jgr.2021.03.009)
Supplement: Multimedia component 1 [file mmc1.docx]

Supplementary materials

**Korean red ginseng** **exerts anti-inflammatory and autophagy-promoting activities in aged mice**

**Jin Kyeong Kim ^1^, Kon Kuk Shin^1^, Haeyeop Kim ^1^, Yo Han Hong ^1^, Wooram Choi ^1^, Yi-Seong Kwak^2^, Chang-Kyun Han ^2^,** **Sun Hee Hyun ^2,**^, Jae Youl Cho ^1,*^**

**^1^** *Department of Integrative Biotechnology, Sungkyunkwan University, Suwon 16419, Republic of Korea*

**^2^** *R&D Headquarters, Korea Ginseng Corporation, Daejeon, Republic of Korea*

**^*^** Corresponding author. Department of Integrative Biotechnology, Sungkyunkwan University, Suwon, 16419, South Korea

**^**^** Corresponding author. R&D Headquarters, Korea Ginseng Corporation, Daejeon, Republic of Korea

Short running title: Role of Korean red ginseng in aged mice

*E-mail addresses:* rosekim95@naver.com (J.K. Kim), shuka337@naver.com (K.K. Shin), rlagoduq7283@naver.com (H. Kim), ghddygks13@naver.com (Y.H. Hong), chwoo1028@naver.com (W. Choi), twostar@kgc.co.kr (Y.-S. Kwak), ckhan@kgc.co.kr (C.-K. Han), shhyun@kgc.co.kr (S.H. Hyun), jaecho@skku.edu (J.Y. Cho)

Supplementary Table 1. Major ingredients (ginsenosides) in KRG-WE

| Ingredient | Amount/g |
| --- | --- |
| Ginsenoside-Rg1 | 5.5 mg |
| Ginsenoside-Rg2 | 5.5 mg |
| Ginsenoside-Rg3 | 5.5 mg |
| Carbohydrates | 0.33 g |

Supplementary Table 2. List of primers used in the present study (mouse species)

| Semi-quantitative RT-PCR | | |
| --- | --- | --- |
| Target | Direction | Sequences (5’ to 3’) |
| IL-8 | Forward | TGC ATG GAC AGT CAT CCA CC |
|  | Reverse | ATG ACA GAC CAC AGA ACG GC |
| IL-1β | Forward | CAGGATGAGGACATGAGCACC |
|  | Reverse | CTCTGCAGACTCAAACTCCAC |
| TNF-α | Forward | TTGACCTCAGCGCTGAGTTG |
|  | Reverse | CCTGTAGCCCACGTCGTAGC |
| MCP-1 | Forward | ACTGAAGCCAGCCAGCTCTCTCTT |
|  | Reverse | ACGGGTCAACTTCACATTCA |
| IL-6 | Forward | GGAAATCGTGGAAATGAG |
|  | Reverse | GCTTAGGCATAACGCACT |
| GAPDH | Forward | ACCACAGTCCATGCCATCAC |
|  | Reverse | CCACCACCCTGTTGCTGTAG |
| qRT-PCR | | |
| P50 | Forward | TCCCCTTATCGTCGCCTTTG |
|  | Reverse | GCTCTTGTGCAAACGCATCA |
| P65 | Forward | GCCATAGTTGCGGTCCTTCT |
|  | Reverse | AAGGGCCTGATCCTTCATGG |
| c-Jun | Forward | GCACATCACCACTACACCGA |
|  | Reverse | GGGAAGCGTGTTCTGGCTAT |
| c-Fos | Forward | CGGGTTTCAACGCCGACTAC |
|  | Reverse | AAAGTTGGCACTAGAGACHG |
| ATG7 | Forward | TTGAGCGGCGACAGCATTAG |
|  | Reverse | TTGAGCGGCGACAGCATTAG |
| ATG12 | Forward | TAAACTGGTGGCCTCGGAAC |
|  | Reverse | ATCCCCATGCCTGGGATTTG |
| LC3B | Forward | TTGGTCAAGATCATCCGGCG |
|  | Reverse | AAGCCGAAGGTTTCTTGGGA |
| Beclin-1 | Forward | GCCTCTGAAACTGGACACGA |
|  | Reverse | TAGCCTCTTCCTCCTGGGTC |
| GAPDH | Forward | GGGTCCCAGCTTAGGTTCATC |
|  | Reverse | TACGGCCAAATCCGTTCACA |
